# Safety and Effectiveness of Oral Methylprednisolone Therapy in Comparison With Intramuscular Adrenocorticotropic Hormone and Oral Prednisolone in Children With Infantile Spasms

**DOI:** 10.3389/fneur.2021.756746

**Published:** 2021-12-22

**Authors:** Hong-Min Zhu, Chun-Hui Yuan, Meng-Qing Luo, Xiao-Long Deng, Sheng Huang, Ge-Fei Wu, Jia-Sheng Hu, Cong Yao, Zhi-Sheng Liu

**Affiliations:** ^1^Department of Neurology, Wuhan Children's Hospital, Tongji Medical College, Huazhong University of Science and Technology, Wuhan, China; ^2^Department of Laboratory Medicine, Wuhan Children's Hospital, Tongji Medical College, Huazhong University of Science and Technology, Wuhan, China; ^3^Department of Rehabilitation Medicine, Wuhan First Hospital, Wuhan, China; ^4^Health Care Department, Wuhan Children's Hospital, Tongji Medical College, Huazhong University of Science and Technology, Wuhan, China

**Keywords:** infantile spasms, methylprednisolone, prednisolone, ACTH, electroclinical remission

## Abstract

**Background and Purpose:** To assess the safety and effectiveness of oral methylprednisolone (oMP) in comparison with intramuscular adrenocorticotropic hormone (imACTH) and oral prednisolone (oP) therapies in children with infantile spasms (IS).

**Methods:** In this prospective, open-label, non-blinded, uncontrolled observational study, children (aged 2–24 months) with newly diagnosed IS presenting with hypsarrhythmia or its variants on electroencephalogram (EEG) were included. It was followed by imACTH, oP, or oMP (32–48 mg/day for 2 weeks followed by tapering) treatments. Electroclinical remission/spasm control, relapse, and adverse effects were evaluated in the short-term (days 14 and 42) and intermediary-term (3, 6, and 12 months) intervals.

**Results:** A total of 320 pediatric patients were enrolled: 108, 107, and 105 in the imACTH, oMP, and oP groups, respectively. The proportion of children achieving electroclinical remission on days 14 and 42 was similar among the three groups (day 14: 53.70 vs. 60.75 vs. 51.43%, *p* = 0.362; day 42: 57.55 vs. 63.46 vs. 55.34%, *p* = 0.470). The time to response was significantly faster in the oMP group (6.5 [3.00, 10.00] days vs. 8.00 [5.00, 11.00] days for imACTH and 8.00 [5.00, 13.00] days for oP, *p* = 0.025). Spasm control at 3, 6, and 12 months was also similar in the three groups (*P* = 0.775, 0.667, and 0.779). The relapse rate in the imACTH group (24.10%) was lower than oMP (30.77%) and oP groups (33.33%), and the time taken for relapse in the imACTH group (79.00 [56.50, 152.00] days) was longer than oMP (62.50 [38.00, 121.75] days) and oP groups (71.50 [40.00, 99.75] days), but the differences were not statistically significant (*p* = 0.539 and 0.530, respectively). The occurrence of adverse effects was similar among the three groups.

**Conclusions:** The short and intermediary-term efficacy and recurrence rates of oMP are not inferior to those of imACTH and oP for the treatment of IS. Significantly, the time to achieve electroclinical remission with oMP was quicker than that with imACTH and oP. Considering its convenience, affordability, and the absence of irreversible side effects, oMP can serve as a form of first-line treatment for newly diagnosed IS.

## Introduction

Infantile spasm (IS), a subtype of epileptic spasms, is an epileptic encephalopathy and is frequently associated with hypsarrhythmic interictal electroencephalograms (EEG) and psychomotor retardation (1). The incidence of IS is ~0.25–0.43 per 1,000 live births, predominantly occuring between 3 and 12 months of age, and rarely has an onset after 2 years of age (2). Although IS usually resolves after 5 years of age, the timely delivery of effective treatment to improve cognitive outcomes in children with IS is essential, as most early deaths (≤ 5 years) are related to neurological impairments or comorbidities (3–5).

The United Kingdom Infantile Spasms Study suggested that the ideal treatment agent for IS should achieve rapid control of spasms, normalize the EEG changes early, maintain a spasm-free state, and thus contribute to a better intermediary-term developmental outcome (6). Currently accepted first-line medications for IS are hormonal therapies and vigabatrin, supported by outcome measures limited to short-term control of spasms and improvement of EEG (7). Vigabatrin is known to be toxic to the retina and can cause visual field defects in ~20% of treated children (8); thus, several well-designed clinical trials are dedicated to comparing the developmental outcomes of hormonal therapies (9–11).

According to the reports of the Sri Lanka Infantile Spasms Study (SLISS), the two most commonly used forms of hormonal therapies, the intramuscular adrenocorticotropic hormone (imACTH; 40 to 60 IU/every other day) and the oral prednisolone (oP; 40–60 mg/day), exhibit no difference in the short- (28 days) and intermediary-term (12-month interval) control of newly diagnosed IS and can be considered as equal options for first-line treatment (9, 10). However, in terms of price and availability for children with IS from low-resource settings, imACTH treatment is not a clear choice. Additionally, oP is frequently associated with side effects such as irritability, and its efficacy is ~40–50%, presumably due to late presentation and predominantly symptomatic etiology (12). Currently, corticosteroids, including oP, pulse intravenous methylprednisolone (ivMP), and dexamethasone are the options being used and evaluated for IS treatment, and the search for relatively cheaper alternatives with similar efficacy is ongoing (12–15).

The reported efficacy of ivMP (30 mg/kg/day) in children with IS is ~60–83%, with freedom from spasms being achieved very early (2–3 days following treatment) among responders (11, 13). Recently, oral methylprednisolone (oMP) at least 500 mg/day/5 days is recommended for treating MS relapses, and its efficiency is not inferior to that of ivMP (16–18). Furthermore, adjunctive oMP (1.6 mg/kg/day for 3 days) also reduced the occurrence or severity of renal scarring after acute pyelonephritis in hospitalized children who had a high risk of renal scar formation (19). Hence, we hypothesized that oMP may be beneficial and effective for children with IS, and the current comparative study was undertaken to investigate the efficacy (short- and intermediary-term) and safety of oMP in IS compared with the standard imACTH and oP therapies.

## Methods

### Sample Selection

This study was designed as a prospective, open-label, non-blinded, uncontrolled observational study to evaluate the efficacy and tolerability of oMP in children with newly diagnosed IS. From January 2016 to October 2020, children aged 2–24 months with newly diagnosed epileptic spasms in clusters with electroencephalographic evidence of hypsarrhythmia or its variants were enrolled and assessed for eligibility in the Department of Neurology of Wuhan Children's Hospital. Children with a diagnosis of IS based on the definition proposed by Lux et al. (3) and those who did not receive any previous hormone or antiseizure medications were included. We excluded patients aged <2 or >24 months, those who received previous treatment with contraindications of hormonal therapy, those with tuberous sclerosis complex (TSC)-associated IS, and those with progressive renal, pulmonary, cardiac, or hepatic dysfunction or severe malnutrition. In addition, patients were also excluded if their parents or guardians did not provide proxy consent or could not monitor their response to therapy. Written informed consent was obtained from all parents. Ethics approval was reviewed and approved by the Medical Ethical Committee of Wuhan Children's Hospital, Huazhong University of Science and Technology (2015019).

### Procedures

Before treatment allocation, the baseline characteristics of all eligible participants were assessed using detailed clinical histories and examinations. Participants' ages at onset, perinatal details, family histories, and developmental statuses were recorded. The results of investigations such as routine blood tests, liver and kidney function, blood electrolytes, arterial blood gas, blood lactate, blood ammonia, urine ketones, blood tandem mass spectrometry, neuroimaging (brain computed tomography, and magnetic resonance imaging), EEG, metabolic testing, and Gesell development schedules test were documented. The etiology of IS was classified according to six etiologic categories defined by the International League Against Epilepsy Task Force, including structural, genetic, infectious, metabolic, immune, and unknown (20).

The parents or guardians of all eligible participants were provided with detailed information about the standard treatment options available for IS, including the total cost, efficacy, and potential adverse effects of imACTH, oP, and oMP therapies. They were asked to make an informed choice based on the feasibility and expense of the three treatments. A chest X-ray and tuberculin test were performed to screen for tuberculosis before starting hormonal therapy. The baseline weight and blood pressure were measured, and pyridoxine was administered to exclude pyridoxine-dependent seizures.

The imACTH group received ACTH (SPH No.1 Biochemical & Pharmaceutical Co., LTD) intramuscularly (25 IU/day) for 2 weeks. If spasms continued until day 7 or reappeared from days 8 to 14, the dose was increased to 40 IU/day. After 2 weeks of treatment, all children received oral prednisolone (soluble tablets, China Resources Zizhu Pharmaceutical Company) at an initial 2 mg/kg dose, which was tapered off and stopped over 4 weeks. The oP group received oral prednisolone at a dose of 10 mg four times/day for 2 weeks. If spasms continued until day 7 or reappeared from days 8 to 14, the dose was increased to 20 mg three times/day. Prednisolone was tapered and stopped over 4 weeks: reductions were made once a week, with 30 mg daily, then 20 mg, then 10 mg, and then 5 mg; if on the higher dose of treatment, the doses were 40 mg daily, then 20 mg, then 10 mg, and then 5 mg.

The oMP group received oral methylprednisolone (soluble tablets, PfizerItalias.r.l) at a dose of 8 mg four times/day for 2 weeks. If spasms continued until day 7 or reappeared from day 8 to day 14, the dose was increased to 16 mg three times/day. After 2 weeks of treatment, methylprednisolone was tapered and stopped over 4 weeks: reductions were made once a week, with 24 mg daily, then 16 mg, then 8 mg, and then 4 mg; if on the higher dose of treatment, the doses were 32 mg daily, then 16 mg, then 8 mg, and then 4 mg.

Currently, there are no guidelines or expert consensus for the subsequent treatment of IS following hormone therapy. In this work, all patients received oral topiramate (soluble tablets, Xi'an Janssen Company, China) after the end of 6 weeks of hormone therapy.

After the initial treatment, patients continued to be followed up at different time points. The initial follow-up included reviews at 14 and 42 days posttreatment, followed by subsequent reviews at 3, 6, and 12 months posttreatment. The first two time points were considered markers of a short-term response, and the three later time points were considered markers of an intermediary-term response. Parents or guardians were trained to document spasms and maintain seizure diaries. At all follow-up visits, the children were evaluated by the same pediatric neurologist. Those who relapsed were treated again with hormonal therapies or other oral anticonvulsants at their parents' discretion. Drug compliance was monitored by asking the parents or guardians directly. The numbers of patients who did not present for follow-up and those who died during follow-up were noted separately for the different time points.

### Outcome Evaluation

The primary outcomes measured were the short- and intermediary-term responses. The evaluated outcome indicators for short-term response were the number of children with electroclinical remission (defined as the absence of witnessed spasms for a minimum of 48 h and absence of hypsarrhythmia on a 2-h digital sleep electroencephalograph) at the time of review on days 14 and 42.

The evaluated outcome indicators for intermediary-term response were the number of children with spasm control (defined as the absence of witnessed spasms for ≥28 consecutive days from the time of the last witnessed spasm) at 3, 6, and 12 months after initial therapy.

The secondary outcomes measured were the time to response (defined as the first day after initiation of treatment on which spasms were not seen) and relapse (defined as the recurrence of one or more clusters of spasms in a child who showed initial remission at end of the 2 weeks of treatment), and the time taken for relapse (defined as the number of days from electroclinical remission to spasm recurrence).

Adverse effects (defined as any untoward or unintended responses) were thought to be related to the treatments. The anticipated potential adverse reactions included cushingoid features, weight gain, increased appetite, frequent crying spells, irritability, infections, drowsiness, gastrointestinal upset (vomiting, gastritis, and abdominal pain), and electrolyte imbalances. Other parental concerns regarding side effects were also noted.

### Statistical Analysis

Categorical data are summarized in proportions, and quantitative data are presented as medians and quartiles. The significance of the differences in electroclinical remission and relapses between the three treatment groups were assessed at 14 and 42 days and 3-, 6-, and 12-month intervals using the Chi-square test. The effect size was calculated using the risk ratio (RR; the proportion with outcomes in the methylprednisolone group divided by the proportion with outcomes in the ACTH group) and absolute risk reduction (ARR; the difference of the proportion with outcomes between treatment groups) with 95% confidence intervals (CIs). The number needed to treat (NNT) was calculated based on the number of patients who needed methylprednisolone to achieve spasm remission, compared with a patient who did not. The Kaplan–Meier survival method was used to plot the relapse and response rates under different groups. The significance level was set at 0.05, and all tests were two-tailed. Statistical analyses were performed using SPSS version 22.0.

### Ethics Statement

This study was reviewed and approved by the Medical Ethics Committee of Wuhan Children's Hospital, Tongji Medical College, Huazhong University of Science & Technology (2015019). The parents of the patients signed written informed consent, agreed with their children's participation in this study, and allowed the use of the relevant data and information for scientific research.

## Results

### Baseline Characteristics

A total of 335 children with clinically confirmed epileptic spasms were evaluated. Eventually, only 320 children were included in the study to compose the three treatment groups. A total of 108, 107, and 105 children were allocated to the imACTH, oMP, and oP groups, respectively. The baseline characteristics of the three groups showed no significant differences (Table 1).

**Table 1:** Baseline characteristics of children with IS.

**Baseline characteristics**	**Intramuscular ACTH** **(*n* = 108)**	**Oral methylprednisolone** **(*n* = 107)**	**Oral prednisolone** **(*n* = 105)**	***P* value**
Sex (male, %)	77 (71.30)	77 (71.96)	64 (60.95)	0.156
Age at onset (mon, Median, IQR)	5.20 [4.00, 7.67]	6.00 [4.23, 7.23]	5.50 [3.33, 7.45]	0.443
Age at presentation (mon, Median, IQR)	7.00 [5.22, 9.92]	7.50 [5.67, 9.73]	6.93 [5.10, 9.18]	0.550
Lead time to treatment (mon, Median, IQR)	1.33 [0.64, 2.50]	1.07 [0.57, 2.17]	1.20 [0.53, 2.67]	0.611
Etiology (*n*, %)				0.839
Structural	21 (19.44)	22 (20.56)	22 (20.95)	
Genetic	14 (12.96)	10 (9.35)	12 (11.43)	
Metabolic	1 (0.93)	0 (0.00)	0 (0.00)	
Unknown	72 (66.67)	75 (70.09)	71 (67.62)	
EEG at presentation (*n*, %)				0.676
Hypsarrythmia	71 (65.74)	76 (71.03)	70 (66.67)	
Hypsarrythmia variant	37 (34.26)	31 (28.97)	35 (33.33)	
Gesell Developmental Scales at presentation (*n*, %)				0.350
Normal (DQ ≥ 70)	6 (5.56)	9 (8.41)	14 (13.33)	
Mild (50 ≤ DQ <70)	28 (25.93)	34 (31.78)	26 (24.76)	
Moderate (30 ≤ DQ <50)	41 (37.96)	34 (31.78)	30 (28.57)	
Severe (DQ <30)	33 (30.56)	30 (28.04)	35 (33.33)	

*IS, infantile spasms; IQR, interquartile range; ACTH, adrenocorticotropic hormone*.

**Figure 1:**
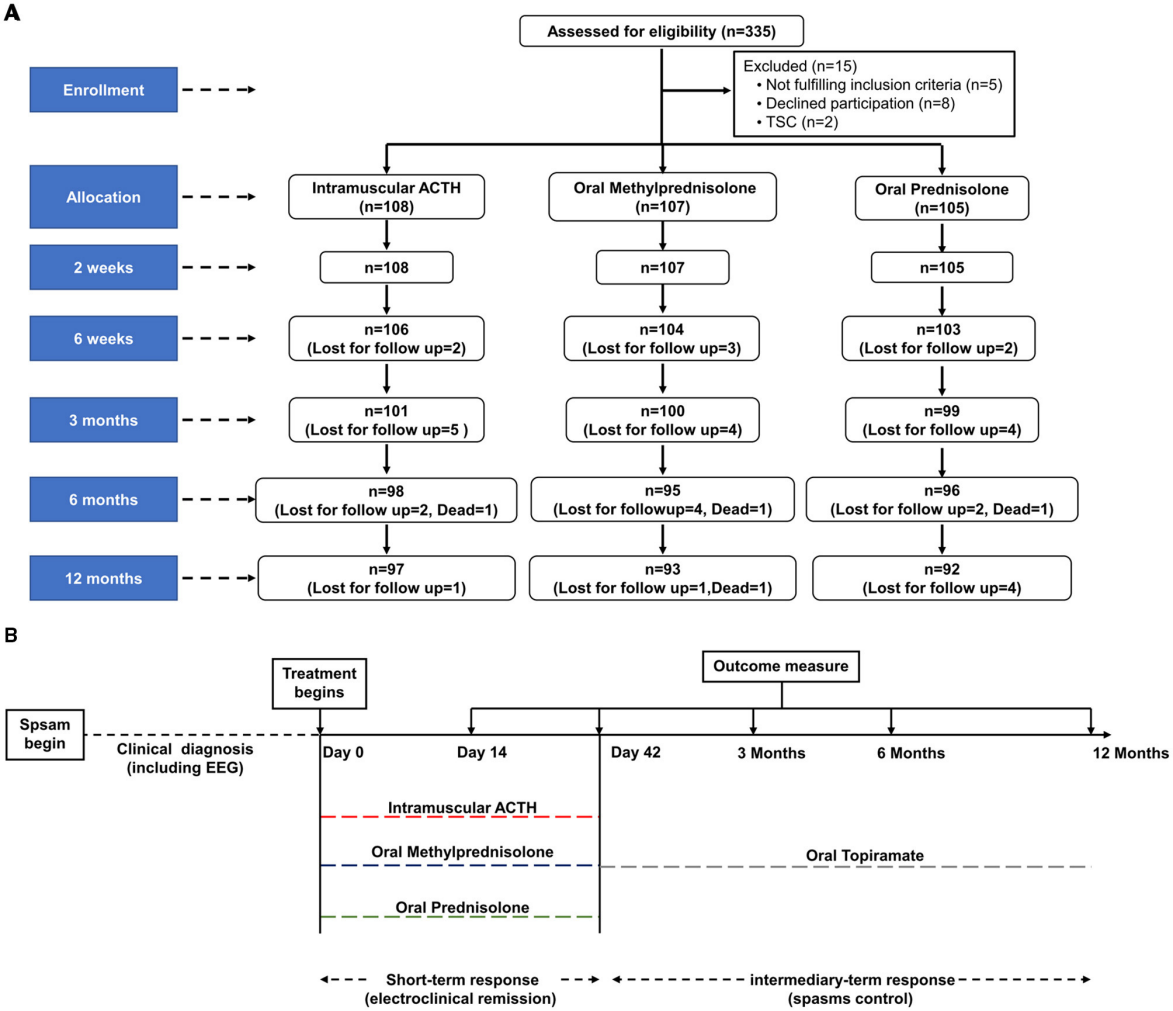
Trial profile and design for assessment of clinical outcome in children with IS. **(A)** Study flow of enrollment, treatment allocation, and follow-up of children with IS. **(B)** Time course of treatment and outcome evaluation. Relapsed pediatric patients were treated again with hormonal therapies or other oral anticonvulsants according to their parents' discretion. EEG, electroencephalogram; ACTH, adrenocorticotropic hormone; IS, infantile spasms.

Details of enrollment, treatment allocation, and follow-up (including the total number of children lost to follow-up or who died during the follow-up period) are outlined in Figure 1A. A total of 282 children completed the follow-up at 12 months. The number of subjects who were lost to follow-up was not statistically different between the three intervention groups: at 42 days (*p* = 0.869), 3 (*p* = 0.801), 6 (*p* = 0.685), and 12 months (*p* = 0.791). Four deaths occurred during the 12-month follow-up, all of which occurred after completing the 6-week initial treatment and were related to other systemic diseases.

### Evaluation of Short-Term Outcomes

The time course of treatment and outcome evaluation is presented in Figure 1B, and the proportions of children with clinical remission at the different time points are summarized in Table 2. On day 14 following initial treatment, electroclinical remission occurred in 58 children in the imACTH group (58/108, 53.70%), 65 children in the oMP group (65/107, 60.75%), and 54 children in the oP group (54/105, 51.43%). There was no statistically significant difference among the three groups (*p* = 0.362). Electroclinical remission by the end of day 42 was higher in the oMP group (66/104, 63.46%) than in the imACTH (61/106, 57.55%) and oP groups (57/103, 55.34%), although the difference was not statistically significant (*p* = 0.470).

**Table 2:** Control of spasms in the followed-up of children with IS after initial treatment.

**Outcome measures**	**Intramuscular ACTH**	**Oral methylprednisolone**	**Oral prednisolone**	***P* value**	**Methylprednisolone vs ACTH**		
					**RR (95%CI)**	**ARR (95%CI)**	**NNT (95%CI)**
Electroclinical remission on 14th days (*n*, %)	58/108 (53.70)	65/107 (60.75)	54/105 (51.43)	0.362	1.18 (0.86, 1.61)	0.07 (-0.06, 0.20)	14 (NNTB5 ∞ NNTH16)
Electroclinical remission on 42th days (*n*, %)	61/106 (57.55)	66/104 (63.46)	57/103 (55.34)	0.470	1.16 (0.83, 1.63)	0.06 (-0.07, 0.19)	17 (NNTB5 ∞ NNTH14)
Spasm control at 3 months (*n*, %)	59/101 (58.42)	57/100 (57.00)	53/99 (53.54)	0.775	0.97 (0.7, 1.34)	−0.01 (-0.15, 0.12)	NNTH70 (NNTB7 ∞ NNTH9)
Spasm control at 6 months (*n*, %)	51/98 (52.04)	48/95 (50.53)	44/96 (45.83)	0.667	0.97 (0.73, 1.29)	−0.02 (-0.16, 0.13)	NNTH66 (NNTB8 ∞ NNTH6)
Spasm control at 12 months (*n*, %)	48/97 (49.48)	45/93 (48.39)	41/92 (44.57)	0.779	0.98 (0.74, 1.29)	−0.01 (-0.15, 0.13)	NNTH91 (NNTB8 ∞ NNTH7)
Time to response (d, IQR)	8.00 [5.00, 11.00]	6.5 [3.00, 10.00]	8 [5.00, 13.00]	0.025			
Relapse (*n*, %)	14/58 (24.10)	20/65 (30.77)	18/54 (33.33)	0.539			
Time taken for relapse (d, IQR)	79.00 [56.50, 152.00]	62.50 [38.00, 121.75]	71.50 [40.00, 99.75]	0.530			

*IS, infantile spasms; IQR, Interquartile range; ACTH, adrenocorticotropic hormone; RR, risk ratio; ARR, absolute risk reduction; NTT, number needed to treat; NNTB, number need to treat to benefit; NNTH, number need to treat to harm*.

**Figure 2:**
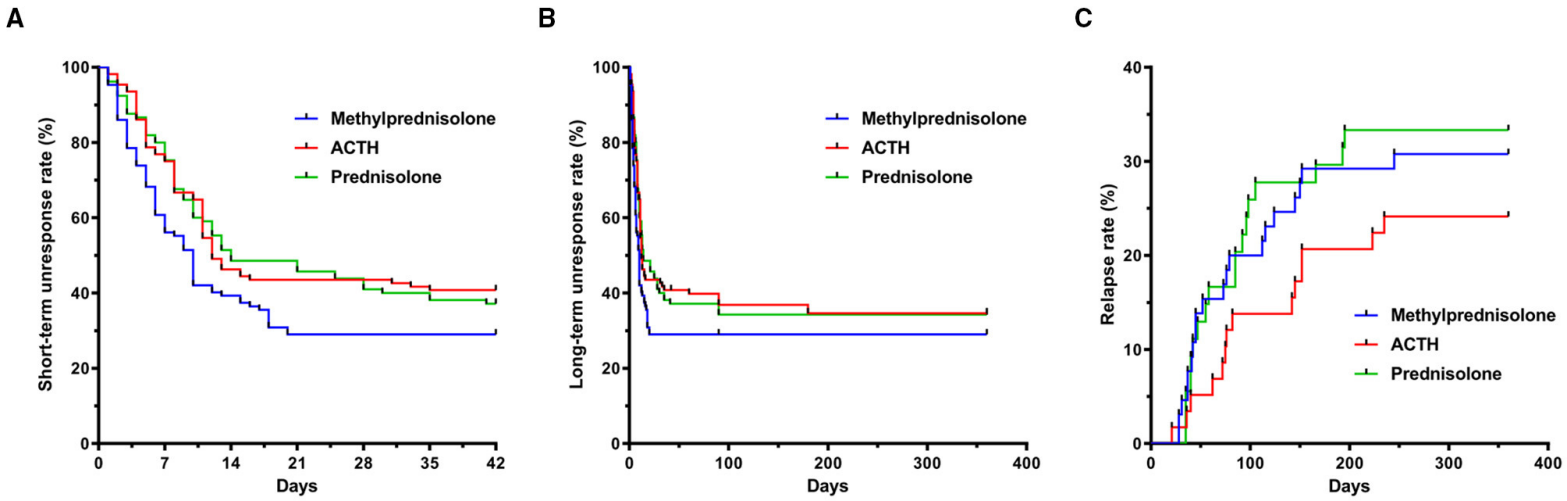
Rates of spasm cessation and relapse of children with IS. **(A)** Unresponsive rate of spasm cessation in imACTH, oP and oMP groups during short-term follow up (42 days interval). **(B)** Unresponsive rate of spasm cessation during intermediary-term follow up (12-month interval). **(C)** Relapse rate in imACTH, oP and oMP groups during intermediary-term follow up (12-month interval). IS, infantile spasms; imACTH, intramuscular adrenocorticotropic hormone; oP, oral prednisolone; oMP, oral methylprednisolone.

The total number of days required for electroclinical remission was significantly lower in children treated with oMP (6.5 days, [3, 10]) compared with those treated with imACTH (8.0 days, [5, 11]) and oP (8.0 days, [5, 13]) (*P* = 0.025). The proportion of infants with an electroclinical remission and the corresponding required days within 42 consecutive days post initial treatment is graphically presented in Figure 2A.

### Evaluation of Intermediary-Term Outcomes

The number of children with spasm control at 3 months in the imACTH (59/101, 58.42%), oMP (57/100, 57.00%), and oP (53/99, 53.54%) groups showed no significant difference (*P* = 0.775). In addition, the proportions of children in whom spasm control was achieved also showed no significant difference at 6 (*p* = 0.667) and 12 months (*p* = 0.779) in the three groups (Table 2; Figure 2B).

After hormonal treatment, the total relapse number in children with spasm control was 52 (52/177, 29.38%) during the 12-month follow-up period (Table 2). Although the relapse rate in the imACTH group (14/58, 24.1%) was relatively lower than that in the oMP (20/65, 30.77%) and oP groups (18/54, 33.33%), this difference was not statistically significant (*p* = 0.539). In addition, no significant difference was noted in the time taken for relapse within each treatment group (*p* = 0.530), although children within the oMP group (62.50, [38.00, 121.75]) tended to relapse earlier than those in the imACTH (79.00, [56.50, 152.00]) and oP groups (71.50, [40.00, 99.75]; Table 2; Figure 2C).

### Tolerability of Adverse Events

Adverse effects observed in the three groups included cushingoid features, weight gain, increased appetite, frequent crying spells, irritability, drowsiness, increased susceptibility to infection, gastrointestinal upset (vomiting, gastritis, and abdominal pain), and electrolyte imbalances (Table 3). The most common adverse effects in the three groups were cushingoid features, increased appetite, and weight gain, which are known reversible side effects of prolonged steroid therapy. However, there was no statistically significant difference in adverse effects among the three groups.

**Table 3:** Comparison of side effects in children with IS.

**Side effects**	**Intramuscular ACTH** **(*n* = 108)**	**Oral prednisolone** **(*n* = 107)**	**Oral methylprednisolone** **(*n* = 105)**	***P* value**
Cushingoid features, *n* (%)	53 (49.07)	51 (47.66)	47 (44.76)	0.814
Weight gain, *n* (%)	36 (33.33)	41 (38.32)	35 (33.33)	0.678
Increased appetite, *n* (%)	45 (41.67)	52 (48.60)	47 (44.76)	0.593
Frequent crying spells, *n* (%)	27 (25.00)	33 (30.84)	36 (34.29)	0.326
Irritability, *n* (%)	13 (12.04)	8 (7.48)	11 (10.48)	0.527
Drowsiness, *n* (%)	7 (6.48)	5 (4.67)	9 (8.57)	0.518
Increased susceptibility to infection, *n* (%)	8 (7.41)	6 (5.61)	5 (4.76)	0.705
Gastrointestinal upset, *n* (%)	5 (4.63)	8 (7.48)	3 (2.86)	0.297
Electrolyte imbalances, *n* (%)	2 (1.85)	1 (0.93)	1 (0.95)	0.787

*IS, infantile spasms; IQR, interquartile range; ACTH, adrenocorticotropic hormone*.

## Discussion

The most accepted standard first-line treatment for IS is hormonal therapy, which mainly refers to imACTH and oP. ACTH is preferred but is expensive and difficult to obtain in some countries. Several trials and meta-analyses have confirmed that high-dose oP (40–60 mg/day) is not inferior to ACTH and is a more cost-effective treatment for IS (16, 17, 21, 22). However, the response time to spasm control, duration, and the relapse rate varied widely across studies (16, 17, 23). Thus, we conducted an open-label observational study to investigate the effectiveness of oMP, imACTH, and oP for the treatment of IS. One important reason for the variable response rates is the differences in the underlying etiologies (i.e., structural, metabolic, and genetic), as Japanese children respond well to pyridoxine when administered as a first line therapy (24, 25). The large prospective nature of this study ensured that the baseline characteristics were comparable among the three groups, which enabled greater power to distinguish the effectiveness of different treatment options.

In terms of short- and intermediary-term spasm cessation, high-dose oP and imACTH have been confirmed to show no significant difference in response rate; the inconsistency regarding these treatments refers to the time until spasm cessation (16, 17, 21). Wirrell et al. (23) reported that high-dose ACTH (>140 IU/m^2^/day) was associated with a greater early response rate than oP by analyzing the multicenter database of the National Infantile Spasms Consortium, whereas Wanigasinghe et al. found that the time required for spasm cessation was quicker in children treated with oP compared with imACTH at a dose of 40–60 IU every other day (3.85 vs. 8.65 days) in the SLISS trial. In this study, the total number of days required for spasm control was ~8 days in children treated with oP and imACTH (25–40 IU/d). In addition, oP and imACTH showed no significant difference in the time until a response in 3/5 randomized controlled trials, and the mean time to spasm cessation ranged from 3 to 8 days in a metaanalysis conducted by Kuo et al. (21). The lead time to treatment affects intermediary-term neurodevelopmental outcomes (6).

The time from spasm onset to initiation of treatment was 15 days in a study conducted by Wirrell et al. (23) and ~1 month in our current study. Thus, we believe that the difference in the time until spasm cessation may have resulted from the variability of the lead time to treatment and the dose of hormones in individual studies. Children with IS should receive hormone therapy as soon as possible.

Prednisolone displays non-linear plasma protein binding in the therapeutic range, whereas methylprednisolone concentrations are proportional to dose, and no determination of plasma protein binding is needed (26). Recently, oMP has been recommended for treating MS relapses, and its efficiency is not inferior to that of ivMP (16–18). In this work, the response rate of oMP exhibited no significant difference with high-dose oP and imACTH, but the time required for spasm control was quicker (6.5 days, [3,10]) at a dose of 8 mg four times/day or 16 mg three times/day (~4–8 mg/kg/day). oMP at a dose of 625 mg/day (~12.5 mg/kg/day) exhibits comparable effectiveness and may not be inferior to the standard-high-dose (1,250 mg/day, ~25 mg/kg/day) for adult MS relapse (18). In this work, the oral oMP dose was moderate to high; and the dose of oMP was calculated to the equal dose of oral prednisolone (oMP 4 mg = oP 5 mg), which considered as equal options for first-line treatment of IS. Since oMP has no consensus statement for IS treatment, the optimized dose and course of oMP still need to be determined in future.

Due to of their shorter follow-up periods, most studies did not account for relapse rates, and only intermediary-term follow-up was conducted by Wanigasinghe et al. (9, 10, 27), and the relapse rates were 21.4 and 44.4% for oP and imACTH, respectively, in a 12-month interval. In this study, relapses occurred with all treatments during the 12-month follow-up period but were more frequent with oP (18/54, 33.33%) and oMP (20/65, 30.77%); the relapse rate in the imACTH group was only 24.1% (14/58). The increased relapse rate of oP compared to imACTH (24 vs. 18%) was further observed in a 3-month follow-up study conducted by Wirrell et al. (23). The difference in relapse rate may be due to the small sample size (*n* = 97) of the SLISS trial conducted by Wanigasinghe et al. (9, 10, 27), which may lead to overestimating oP's effectiveness. Furthermore, children who received oMP (62.50, [38.00, 121.75]) tended to relapse earlier than those who received imACTH (79.00, [56.50, 152.00]) and oP (71.50, [40.00, 99.75]). The potential reason may be that the plasma clearance of methylprednisolone is faster than that of prednisolone, and ACTH usually becomes effective slowly but its effect lasts longer (28).

The major disadvantage of ivMP for treating IS is the lower rate of sustained electroclinical remission at 6 weeks compared with oP (11, 13), demonstrating the lack of prolonged action of ivMP on IS. However, no such finding was observed in this work, and the intermediary-term efficacy of oMP was comparable to that of oP and imACTH. The potential reason may be related to the long period of relatively high doses of oMP used in the current study, while ivMP was pulsed for only 3 days followed by oP for 2–4 weeks. oMP and oP are considerably less expensive, more available and convenient options in resource-limited settings compared with imACTH. In combination with the advantage of achieving electroclinical remission earlier than oP, oMP represents a novel treatment option for IS.

The main adverse effects of steroid therapies include increased risk of severe and fatal infections, hypertension, bone loss, glucose intolerance, Cushing syndrome, growth velocity suppression, global cerebral atrophy, and cognitive impairment (28–30). In this study, there was no statistically significant difference in adverse effects among the three groups. As the reversible side effects of prolonged steroid therapy are known (31, 32), none of the children stopped treatment due to side effects, and side effects gradually disappeared when hormone treatments were discontinued, whereas increased appetite and gastrointestinal upset were more common with oP. In addition, the cost of imACTH regimen is ~40 times of oMP in low-setting regions. These results suggest that oMP is well tolerated and can be considered a safe and effective alternative treatment for IS.

Although this study supports the effectiveness of oMP for the treatment of IS, it has some limitations. First, as this is a non-blinded, uncontrolled observational study, complete randomization and blindness cannot be achieved, which will impact the study's efficacy. Second, the reliance on parental reporting of spasms may not be accurate without performing 24-h EEG to substantiate complete cessation of spasms. Lastly, both imACTH and ivMP therapies had limited effectiveness in IS with structural etiologies (14), and the association of response rates with the differences in the underlying etiologies was not assessed because of the large number of subjects in the unknown category (66.67–70.09%).

In conclusion, the highlight of this work is that the short- and intermediary-term efficacy and recurrence rate of oMP were not inferior to those of imACTH and oP, which are the standard first-line treatments for IS. More importantly, the time to achieve spasm cessation of oMP was lower than that of imACTH and oP. This should guide pediatricians worldwide, especially in regions where ACTH or vigabatrin remains unavailable, in choosing to use oMP as a form of first-line treatment for newly diagnosed IS. The impact of this finding is enormous, particularly concerning the convenience, affordability, and lack of irreversible side effects of oMP.

## Data Availability Statement

The original contributions presented in the study are included in the article/Supplementary Material, further inquiries can be directed to the corresponding authors.

## Ethics Statement

The studies involving human participants were reviewed and approved by Medical Ethics Committee of Wuhan Children's Hospital, Tongji Medical College, Huazhong University of Science & Technology (2015019). Written informed consent to participate in this study was provided by the participants' legal guardian/next of kin.

## Author Contributions

Z-SL, CY, J-SH, and G-FW: study conception and design. H-MZ, M-QL, SH, and X-LD: data acquisition. C-HY, CY, and H-MZ: analysis and data interpretation. H-MZ and C-HY: drafting of the manuscript. All authors contributed to the article and approved the submitted version.

## Funding

This work was supported by the Wuhan Municipal Health Commission of Clinical Research Projects (NO. WX17B11).

## Conflict of Interest

The authors declare that the research was conducted in the absence of any commercial or financial relationships that could be construed as a potential conflict of interest.

## Publisher's Note

All claims expressed in this article are solely those of the authors and do not necessarily represent those of their affiliated organizations, or those of the publisher, the editors and the reviewers. Any product that may be evaluated in this article, or claim that may be made by its manufacturer, is not guaranteed or endorsed by the publisher.
